# Mobile Health–Supported Active Syndrome Surveillance for COVID-19 Early Case Finding in Addis Ababa, Ethiopia: Comparative Study

**DOI:** 10.2196/43492

**Published:** 2023-08-28

**Authors:** Haileleul Bisrat, Tsegahun Manyazewal, Abebaw Fekadu

**Affiliations:** 1 Center for Innovative Drug Development and Therapeutic Trials for Africa (CDT-Africa) College of Health Sciences Addis Ababa University Addis Abeba Ethiopia

**Keywords:** mobile health, mHealth, digital health, COVID-19, syndrome assessment, surveillance, Ethiopia, public health, syndrome surveillance, self-care, telemedicine, telecom, SARS-CoV-2

## Abstract

**Background:**

Since most people in low-income countries do not have access to reliable laboratory services, early diagnosis of life-threatening diseases like COVID-19 remains challenging. Facilitating real-time assessment of the health status in a given population, mobile health (mHealth)–supported syndrome surveillance might help identify disease conditions earlier and save lives cost-effectively.

**Objective:**

This study aimed to evaluate the potential use of mHealth-supported active syndrome surveillance for COVID-19 early case finding in Addis Ababa, Ethiopia.

**Methods:**

A comparative cross-sectional study was conducted among adults randomly selected from the Ethio telecom list of mobile phone numbers. Participants underwent a comprehensive phone interview for COVID-19 syndromic assessments, and their symptoms were scored and interpreted based on national guidelines. Participants who exhibited COVID-19 syndromes were advised to have COVID-19 diagnostic testing at nearby health care facilities and seek treatment accordingly. Participants were asked about their test results, and these were cross-checked against the actual facility-based data. Estimates of COVID-19 detection by mHealth-supported syndromic assessments and facility-based tests were compared using Cohen Kappa (κ), the receiver operating characteristic curve, sensitivity, and specificity analysis.

**Results:**

A total of 2741 adults (n=1476, 53.8% men and n=1265, 46.2% women) were interviewed through the mHealth platform during the period from December 2021 to February 2022. Among them, 1371 (50%) had COVID-19 symptoms at least once and underwent facility-based COVID-19 diagnostic testing as self-reported, with 884 (64.5%) confirmed cases recorded in facility-based registries. The syndrome assessment model had an optimal likelihood cut-off point sensitivity of 46% (95% CI 38.4-54.6) and specificity of 98% (95% CI 96.7-98.9). The area under the receiver operating characteristic curve was 0.87 (95% CI 0.83-0.91). The level of agreement between the mHealth-supported syndrome assessment and the COVID-19 test results was moderate (κ=0.54, 95% CI 0.46-0.60).

**Conclusions:**

In this study, the level of agreement between the mHealth-supported syndromic assessment and the actual laboratory-confirmed results for COVID-19 was found to be reasonable, at 89%. The mHealth-supported syndromic assessment of COVID-19 represents a potential alternative method to the standard laboratory-based confirmatory diagnosis, enabling the early detection of COVID-19 cases in hard-to-reach communities, and informing patients about self-care and disease management in a cost-effective manner. These findings can guide future research efforts in developing and integrating digital health into continuous active surveillance of emerging infectious diseases.

## Introduction

The global health community learned a lesson from the COVID-19 pandemic: each country needs to strengthen its epidemic preparedness and response capacity to mitigate emerging and reemerging infectious diseases at an earlier stage. It was noted that trajectories of the COVID-19 pandemic would have been curtailed through active and adequate disease surveillance, laboratory infrastructure, health workforce, pandemic planning, and management systems [1,2]. With limited disease preparedness and response capacity and a largely susceptible economy, Africa was endangered by the pandemic and its interrelated economic consequences [3-5]. Although the pandemic progressed more slowly in Africa compared to the rest of the globe, almost all African countries have been affected by the pandemic in one way or another [5-7]. More than 9.5 million COVID-19 confirmed cases were recorded across the African continent as of May 08, 2023 [8].

Ethiopia, a country in sub-Saharan Africa, confirmed its first case of COVID-19 on March 13, 2020. Two days later, the World Health Organization declared a pandemic of the disease [9]. As of May 10, 2023, there have been 500,853 confirmed cases of COVID-19, with 7574 deaths, reported to the World Health Organization [8]. COVID-19 placed a significant burden on patients with chronic diseases in Ethiopia, affecting their ability to access their routine clinical care and treatment [10,11]. Health care workers [12,13] and the community at large [14,15] had uncertainties in implementing preventive measures against the disease. Working with the global health community, the Ethiopian government introduced vaccines against COVID-19, and as of May 6, 2023, the country administered 54,041,862 vaccine doses [9]. Ethiopia is one of the resource-constrained countries, overwhelmed by a double burden of infectious and noninfectious diseases and with limited capacity to find and treat cases at an early stage [15-18]. The country has limited health care infrastructure and workforce [19-21].

The systematic digitalization of the health care industry may improve access to quality care and lower health care costs. Through the use of smartphones, health information technology, wearable devices, telemedicine, and personalized medicine, digital health technologies have the potential to facilitate health care and attain intended health outcomes [22-24]. Mobile health (mHealth) apps are emerging as a strategy to improve health care delivery and outcomes [25-27]. As mobile phones are more accessible to many people in low- and middle-income nations like Ethiopia, the technology is likely a means to offer impactful and affordable solutions to address diseases of significant public health importance.

In the context of COVID-19, the potential use of mHealth technology to prevent and control the pandemic in Africa has been studied [28-32]; however, evidence is limited, especially on the potential impact of mHealth technology on active syndrome surveillance. As the number of mobile phone owners continues to rise in many African countries, including Ethiopia, mHealth technologies may aid in the early identification of COVID-19 cases within the community and link them to diagnostic testing centers.

Africa showed limited capacity and flexibility to scale up COVID-19 testing, effectively tracing contacts of confirmed cases, and promptly training and deploying community health workers to help in the early identification of cases and their connection to appropriate care [33]. Existing conventional surveillance systems were not sufficient enough to respond to the COVID-19 pandemic. Moreover, it was difficult for the majority of the African population to fully comply with the preventive measures due to socioeconomic consequences. Hence, the problem of mitigating COVID-19 spanned across infrastructure, human resources for health, diagnosis, logistics, population literacy, and economy. There was a strong need to develop a simple alternative method to help detect COVID-19 cases in the community quickly and cost-effectively. Digital health interventions offered such potential.

Therefore, we aimed to evaluate the potential use of mHealth-supported active syndrome surveillance for the early identification of COVID-19 cases in Addis Ababa, Ethiopia.

## Methods

### Study Design and Participants

This study is part of an ongoing national, population-based cohort mHealth-supported study—“mHealth-supported continuous national surveillance of COVID-19 for early case finding and population-level impact and control in Ethiopia (EPIC).” This specific study is a population-based cross-sectional comparative study, using mobile phone call surveys and a cross-sectional comparison of mHealth-supported COVID-19 syndrome diagnosis versus confirmatory laboratory tests. In this study, a mobile call survey was deemed an appropriate data collection method, given the nature of the pandemic, the wide geographic area of the country, and economic feasibility.

Eligible participants were adults aged 18 years and older living in Ethiopia, speaking one or more of the 3 Ethiopian working languages (Amharic, Afan Oromo, and Tigrigna), and having no hearing or cognitive impairment or serious mental illness that impedes participation in the study. Potential participants were selected from the population of individuals with mobile phones registered centrally with either the federal or Addis Ababa authorities. Hence, participants were randomly selected from the list of mobile phone numbers available in the country using computer-generated random numbers. Initially, 11 million numbers were generated, from which 30,000 phone numbers were randomly selected. Our study uses the first 4180 phone numbers from the 30,000 randomly generated numbers.

### Data Collection

Participants underwent a comprehensive phone interview for COVID-19 syndromic assessments, and their symptoms were scored and interpreted based on national guidelines. COVID-19–like symptoms were measured using a syndromic assessment for acute respiratory illnesses (fever and at least one sign or symptom of respiratory diseases, such as cough, sore throat, runny nose, shortness of breath, loss of smell, and loss of taste). Participants who had COVID-19 were advised to have COVID-19 diagnostic testing at nearby health care facilities and seek treatment accordingly. Participants were asked about their test results, and these were crosschecked against the actual facility-based data.

Questionnaires were implemented on an electronic data capture platform, Open Data Kit, the free app for Android devices that is used to collect and compile data. Whenever a phone number was not responsive or unanswered during the first attempt, repeated calls were made up to 3 times before excluding it from the study.

Data on COVID-19 laboratory test results were collected by reviewing log charts in hospitals and health centers where the participants had undergone laboratory testing. During the phone interview, those who had been tested and had received the COVID-19 test results, along with other indicators, were cross-checked. From the data gathered from government referrals and specialized hospitals, government health centers, private hospitals, and private clinics, it was possible to determine whether the information provided to the participants who indicated they had undergone a COVID-19 test, along with other indicators, matched the registration logs at health care facilities.

**Figure 1:**
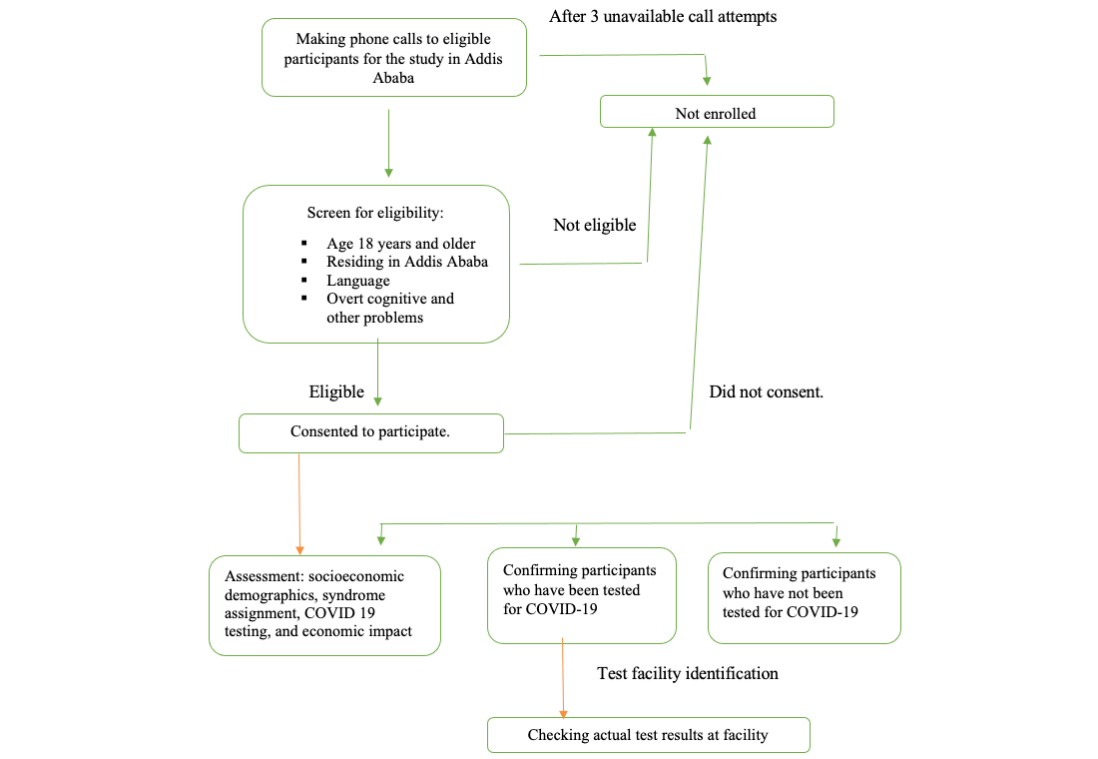
Data collection procedure.

Different quality assurance strategies were followed to ensure data integrity, quality, and reliability. In the data collection process, 13 data collectors with a minimum of BSc degrees in the health profession, along with 1 supervisor, undertook the data collection responsibilities after all contractual and training aspects were finalized. The survey procedures and tools were pretested for utility, feasibility, and acceptability. There was a face-to-face meeting with the supervisor every morning for reviewing and planning purposes. The data were stored daily on a central server at Center for Innovative Drug Development and Therapeutic Trials (CDT)-Africa in Addis Ababa University, Ethiopia. Figure 1 presents the overall procedures followed for data collection.

### Ethics Approval

This study has been reviewed and approved by the Institutional Review Board of the College of Health Sciences at Addis Ababa University (086/20/CDT). The study participants were briefed about the study over the phone and asked about their willingness to participate in the study. They were enrolled only after giving verbal consent. There was no compensation for participation in the study. The data were kept confidential and used for study purposes only.

### Statistical Analysis

Interpretation of syndrome assessment followed the guidelines of the Ethiopian Ministry of Health. COVID-19 and non–COVID-19 signs and symptoms were categorized by assigning a score of 1 to COVID-19 and a score of 0 to Non–COVID-19 suspects. The formula used to determine the distribution of participants based on syndrome assessment and test results is as follows (Table 1):

κ *= P_o_ – P_e_ / 1 – P_e_ = 1 – 1 – P_o_ / 1 – P_e_*

Where *P_o_* = relative observed agreement among raters, *P_e_* = hypothetical probability of chance agreement, and κ = kappa status.

Po = (a + d) / N

Where *a* = the total number of instances that both raters said were correct (ie, the raters are in agreement), *b* = the total number of instances where what rater 2 said was incorrect, but what rater 1 said was correct (ie, rater disagreement), *c* = the total number of instances where what rater 1 said was incorrect, but what rater 2 said was correct (ie, rater disagreement), and *d* = the total number of instances where what both raters said was incorrect (ie, raters are in agreement).

For Cohen kappa, the chance agreement is defined as the sum of the products of marginal distributions, which is as follows:


*P_e_ (κ) = (P.1 P1.) + (P.2 P2.)*

**Table 1:** Determination of the distribution of participants based on syndrome assessment and test results.

Test result	Syndrome assessment			
	Category 1 (yes)	Category 2 (no)	Total	
Category 1 (yes)	a	b	a + b	P1. = (a + b) / N
Category 2 (no)	c	d	c + d	P2. = (c + d) / N
Total	a + c	b + d	N	
	P.1 = (a + c) / N	P.2 = (b + d) / N		

For both the syndrome assessment and test results, the area under the receiver operating characteristic (ROC) curve was used to assess the overall diagnostic performance, indicating the possibility of accurately diagnosing all symptoms. The area under the curve of a procedure should be close to 1 for it to be highly sensitive and specific. The approach is considered more accurate if the curve closely aligns with the left-hand border and the top border of the ROC space. If the area under the ROC curve exceeded 0.75, we deemed our methods to be appropriate.

For validity measures, sensitivity and specificity along with their corresponding 95% CIs were compared between syndrome assessment and laboratory test results. Sensitivity refers to the ability of the COVID-19 syndrome assessment to correctly identify COVID-19 test results as designated by the test outcome, while specificity refers to the proportion of cases caused by other factors, correctly identified as non–COVID-19. These 2 measures are closely related to type 1 and type 2 errors. Hence, both sensitivity and specificity were calculated. The formula for the calculation was defined as follows: sensitivity = *TP / (TP + FN)* and specificity = *TN / (FP + TN)*. In these formulas, TP is true positive, FP is false positive, TN is true negative, and FN is false negative.

Data analysis was conducted using STATA 17 (StataCorp) software. Individual participants had a unique ID, and all data sets were merged using this ID before analysis. Two separate variables, COVID-19 syndrome assessment and COVID-19 laboratory test results, were generated for comparison purposes and for documenting the trend of COVID-19 results. Coding, recording, labeling, and analysis were also done.

Estimates of COVID-19 detection through syndrome assessment and test results were compared using Cohen kappa (κ) and ROC curve analysis. A κ value less than 0 indicates no agreement, and values between 0 and 0.20 indicate slight agreement; values from 0.21 to 0.40 denote fair agreement; values from 0.41 to 0.60 suggest moderate agreement; those from 0.61 to 0.80 imply substantial agreement; and finally, values from 0.81 to 1 indicate an almost perfect agreement [34].

## Results

### Background Characteristics

**Figure 2:**
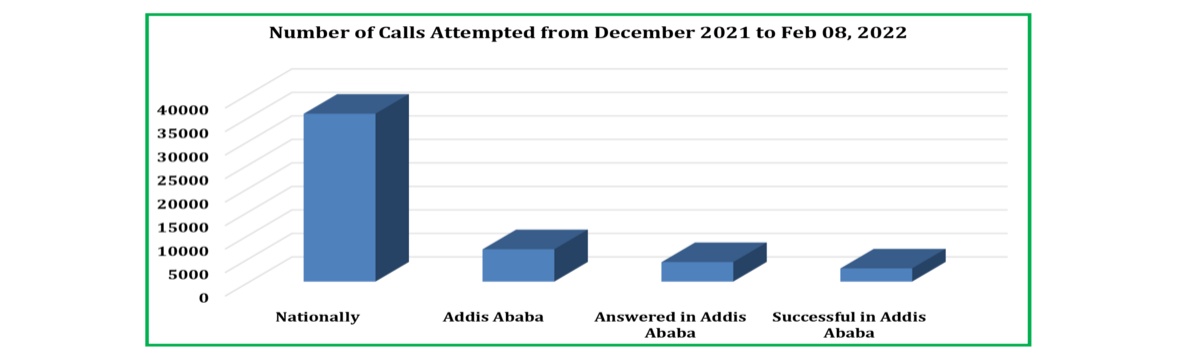
Background characteristics.

In this study, a total of 35,646 national calls were made, of which 2741 participants were from Addis Ababa. Of the 6818 calls made, 4077 were characterized as unavailable, unanswered, switched off, disconnected, or hung up, resulting in a response rate of 67.2% among the participants from Addis Ababa (Figure 2).

A total of 2741 data points were collected through telephone (mobile phone) interviews for the period from December 2021 to February 2022. Of these, 1476 (53.85%) were male and 1265 (46.15%) were female individuals; 1213 (44.25%) were from the age group of 18-29 years, 1151 (41.99%) had a diploma or higher academic qualification, and 1443 (52.65%) were married (Table 2).

**Table 2:** Background characteristics of the study participants (N=2741).

Background characteristics		Values, n (%)
**Sex**		
	Male	1476 (53.85)
	Female	1265 (46.15)
**Age category (years)**		
	18-29	1213 (44.25)
	30-39	879 (32.07)
	40-49	379 (13.83)
	50-59	159 (5.80)
	60-69	78 (2.85)
	70-79	31 (1.13)
	≥80	2 (0.07)
**Level of education**		
	Cannot read or write	75 (2.74)
	Primary school	450 (16.42)
	Secondary school	993 (36.23)
	Certificate	72 (2.63)
	Diploma or above	1151 (41.99)
**Marital status**		
	Single	1162 (42.39)
	Married	1443 (52.65)
	Divorced	86 (3.14)
	Widowed	50 (1.82)

### Comparison of mHealth-Supported Syndrome Assessment Versus Laboratory Test Results

Of the total 2741 data points collected through mobile phones, 1371 participants had undergone a COVID-19 test. Among the tested participants, 884 (64.5%) received confirmation of their results from the respective hospitals and health centers; however, the remaining 487 participants did not have their results recorded in the health care facilities and were consequently excluded from this particular analysis.

There were some variations in laboratory results collected in different months. In December 2021, confirmed COVID-19 cases accounted for 37% (32/86), while results from syndrome assessment constituted only 29% (45/155) of the participants with a positive COVID-19 outcome. In January, confirmed results accounted for 63% (54/86), while syndrome assessment indicated that 67% (489/729) were non–COVID-19 cases. Table 3 summarizes the COVID-19 mHealth-supported syndrome assessment and laboratory test results categorized by months and COVID-19 results.

**Table 3:** COVID-19 mHealth-supported syndrome assessment and laboratory test results by months and test results (N=884).

Month and year	COVID-19 laboratory test result		COVID-19 mHealth-supported syndrome assessment	
	Non–COVID-19 (n=798), n (%)	COVID-19 (n=86), n (%)	Non–COVID-19 (n=729), n (%)	COVID-19 (n=155), n (%)
December 2021	252 (32)	32 (37)	239 (33)	45 (29)
January 2022	544 (68)	54 (63)	489 (67)	109 (70)
February 2022	2 (0.25)	0 (0)	1 (0.14)	1 (1)

### Agreement Between mHealth-Supported Syndrome Assessment and Laboratory Test Results

The observed agreement between the mHealth-supported syndrome assessment and laboratory results was 0.89 and Cohen kappa for the hypothetical probability of chance agreement was 0.54. The syndrome assessment model had an optimal likelihood cut-off point sensitivity of 46% (95% CI 38.4-54.6) and specificity of 98% (95% CI 96.7-98.9; Table 4).

**Table 4:** Distribution of participants by mHealth-supported syndrome assessment and test results for COVID-19 using mHealth categories.

Laboratory test results	mHealth-supported syndrome assessment			
	COVID-19	Non–COVID-19	Total	
COVID-19	72	14	86	P1. = 0.097
Non–COVID-19	83	715	798	P2. = 0.902
Total	155	729	884	
	P.1 = 0.175	P.2 = 0.824		

### ROC analysis

**Figure 3:**
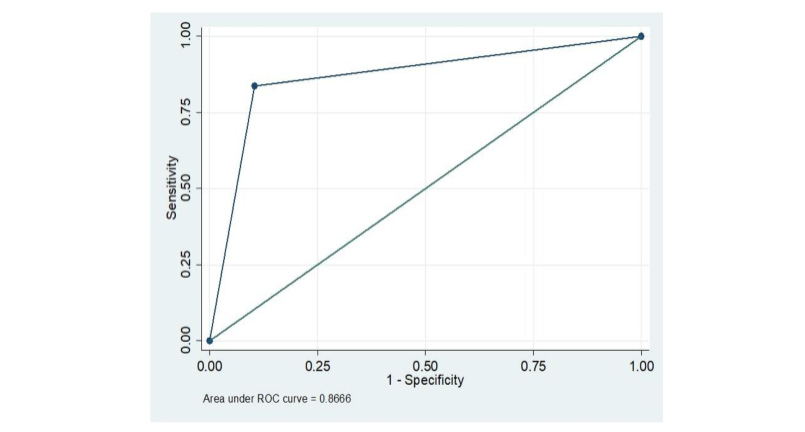
Receiver operating characteristic (ROC) curve.

Figure 3 presents the ROC analysis in a one-to-one square. The area under the curve captures the relationship between the sensitivity and specificity of the COVID-19 test results and reflects the performance of the method used. The curve follows the left-hand border and the top border of the ROC space, indicating an acceptable level of accuracy. The ROC curve shows that the predicted area under the ROC curve for COVID-9 is 0.87 for the mHealth-supported syndrome assessment results compared to the laboratory test results. This indicates the good diagnostics performance of the syndrome assessment method used.

## Discussion

### Principal Findings

In this study, we evaluated the potential use of mHealth-supported active syndrome surveillance for COVID-19 early case finding. Interviewing a total of 2741 adults through the mHealth platform, we found 50% (1371/2741) had COVID-19 symptoms at least once, and 64.5% (884/2741) had laboratory test results recorded in facility-based registries. The syndrome assessment model had an optimal likelihood cut-off point sensitivity and specificity of 46% and 98%, respectively, with an area under the ROC curve of 0.87 and a moderate level of agreement between the mHealth-supported syndrome assessment and the laboratory-confirmed COVID-19 test results (κ=0.54).

One of the efficient methods for addressing epidemic or pandemic outbreaks is the use of health information technologies, such as mHealth, which facilitate remote communication. To manage and regulate the recently emerged COVID-19 pandemic, studies are underway to assess the functionalities of different digital health technologies. This study described the use of mHealth-supported active surveillance for COVID-19 early case findings by evaluating the agreement between the mHealth-supported syndrome assessment and laboratory-confirmed COVID-19 test results. The outcomes revealed a moderate level of agreement between the results of the COVID-19 syndrome assessment supported by mHealth and the laboratory test results, at 89%, with a kappa value of 0.54. This suggests that mHealth could be a potential alternative to the standard laboratory-based confirmatory diagnosis to find COVID-19 cases. Previous studies have shown that mHealth technologies could have significant contributions to self-care for patients with COVID-19 [35] and the dissemination of COVID-19–related information [36,37]. As mHealth and other digital health technologies are yet at an early stage of development in Africa, the capacity and readiness of each country to effectively adopt, implement, and scale up digital health interventions require due diligence [38-42].

The findings of this study show that the mHealth-supported COVID-19 syndrome assessment model has an optimal likelihood cut-off point sensitivity and specificity when compared with the laboratory-based tests. The ROC curve indicates the good diagnostics performance of the mHealth syndrome assessment model. The study used a holistic syndrome assessment approach, based on the national guidelines, to examine and interpret the COVID-19 status of individuals participating in the study. Previous studies show that syndromic diagnosis of COVID-19 based on a single symptom cannot accurately identify individuals who might have the virus, and hence, investigating cases through combinations of syndromes along with additional information, such as recent contacts, travel history, or vaccination status, is necessary [43-45]. Studies show that although digital health–enabled communication may not be as effective as in-person communication, it represents a safe and efficient alternative to collecting evidence-based medical history, especially during the COVID-19 period, when in-person care cannot be provided [46].

Investigating the potential use of mHealth-supported active syndrome surveillance for COVID-19 early case finding in Ethiopia, this study provides important insights. Future studies can further explore how digital health technologies can be used for the early identification of emerging infectious diseases, reducing their transmissions, as well as monitoring and mitigating their undue impact. Our findings inform the scientific community about how mHealth can be adapted for health system responses and the implementation challenges and opportunities within a resource-constrained country context. Scientific evidence regarding the potential use of such digital health technologies and their interrelated challenges is important for guiding policy and practice, especially in countries that have not yet fully embraced digital health interventions.

### Study Limitations

This research has certain limitations. The response rate of the participants was not as anticipated. Because the survey was conducted over the phone, only those who had cell phones at the time of data collection were included in the survey, which limits the generalizability of our findings. Interrupted call connectivity, inconsistency between the participants’ responses and the data in facility registries, and participants undergoing COVID-19 testing for reasons other than syndromes (eg, travel health certificates) were some of the shortcomings in the data collection process. To mitigate these issues, only clear and consistent data were included and used in the analysis and interpretation of the study.

### Conclusions

In this study, the level of agreement between the mHealth-supported syndrome assessment results and the actual laboratory-confirmed COVID-19 results was reasonable at 89%. mHealth-supported syndromic assessment of COVID-19 is a potential alternative method to the standard laboratory-based confirmatory diagnosis to detect COVID-19 cases at an earlier stage in hard-to-reach communities and to advise patients on self-care and disease management in a cost-effective way. The findings show that the mHealth platform is valid for COVID-19 surveillance in Ethiopia, where health infrastructures are limited. Given the growing digital health landscape, the findings of our study offer valuable insight for guiding future research efforts in developing and integrating digital health into continuous active surveillance of emerging infectious diseases.

## Abbreviations

CDT: Center for Innovative Drug Development and Therapeutic Trials
EPIC: early case finding and population-level impact and control in Ethiopia
mHealth: mobile health
ROC: receiver operating characteristic

## Data Availability

The data set supporting the conclusions of this study is included within the paper. Any additional material can be obtained upon reasonable request.
